# COGNITIVE ASSESSMENT OF MARSHALLESE PEOPLE USING QDRS (QUICK DEMENTIA RATING SCALE)

**DOI:** 10.1093/geroni/igae098.3940

**Published:** 2024-12-31

**Authors:** Gohar Azhar, Shakshi Sharma, Sheldon Riklon, Philmar M Kabua, Pearl McElfish, Jeanne Y Wei

**Affiliations:** University of Arkansas for Medical Sciences, Little Rock, Arkansas, United States; University of Arkansas for Medical Sciences, LITTLE ROCK, Arkansas, United States; UAMS Northwest Regional Campus, UAMS Northwest Campus, Arkansas, United States; UAMS Northwest Regional Campus, Fayetteville, Arkansas, United States; UAMS Northwest Regional Campus, Fayetteville, Arkansas, United States; University of Arkansas for Medical Sciences, Little Rock, Arkansas, United States

## Abstract

Health disparities in Marshallese living in the US are common due to language barriers, cultural differences, lack of insurance, and inability to pay. Marshallese face strikingly high rates of heart attacks, diabetes, depression, and anxiety. As cognition in this population has been understudied, we used the Quick Dementia Rating Scale (QDRS), to determine aspects of their cognition. QDRS is a dementia staging tool of 10 items (30 points). Forty-nine subjects consented to the study, which included 34 younger adults (YA) and 15 older adults (OA). Self-reported co-morbidities of diabetes were 67% in OA vs 38% in YA; heart diseases were 67% in OA vs 9% in YA; hypertension was 33% in OA vs 18% in YA; anxiety and depression were 60% in OA vs 26% in YA. Cognitive assessment showed that 13% of OA had moderate to severe difficulty in memory and recall compared to only 2% of YA. Additionally, 7% OA had moderate to severe problems in orientation, attention, concentration, language, and decision-making abilities. Moderately severe problems were also found in behavior with personality changes and reduced personal hygiene in OA. Only 3 % of YA had problems performing tasks or activities outside the home compared to 7% in OA. Furthermore, 13 % of OA had moderate to severe problems with anxiety or depression with no issues reported by YA. Our results demonstrated that higher risks associated with cardiovascular diseases and diabetes mellitus corelated with greater cognitive, behavioral, and functional impairment in older adult Marshallese in Arkansas.

